# Acute Allograft Rejection in Kidney Transplant Recipients Treated With Immune Checkpoint Inhibitors: An Educational Case Report

**DOI:** 10.1177/20543581241289191

**Published:** 2024-10-21

**Authors:** Steven A. Morrison, Amanda J. Vinson

**Affiliations:** 1 Division of Nephrology, Department of Medicine, Dalhousie University, Halifax, NS, Canada

**Keywords:** kidney transplantation, immune checkpoint inhibitor, rejection, immunosuppression

## Abstract

**Rationale:**

Kidney transplant (KT) recipients have an increased risk of malignancy due to chronic immunosuppression. The emerging use of immune checkpoint inhibitors (ICIs) has been a promising development for the treatment of malignancy, but their use adds to the complexity of immunosuppression management for KT recipients. This case report describes 2 cases of acute rejection in KT recipients following ICI initiation and discusses the balance of malignancy treatment with adequate immunosuppression.

**Presenting Concerns of Patients:**

The first patient is a 44-year-old male KT recipient with a diagnosis of metastatic renal cell carcinoma presenting with acute kidney injury 6 days following initiation of an ICI. The second patient is a 73-year-old male KT recipient with a diagnosis of squamous cell carcinoma presenting with acute kidney injury 2 weeks following initiation of an ICI.

**Diagnoses:**

Both patients were diagnosed with acute rejection in the setting of reduced immunosuppression and initiation of an ICI.

**Interventions:**

Both cases received an increased dose of steroid without improvement of graft function. The first patient subsequently underwent a delayed graft nephrectomy due to complications of acute rejection, whereas the second patient did not undergo nephrectomy.

**Outcomes:**

The first patient experienced complications including perioperative bleeding requiring multiple operations, but ultimately stabilized on hemodialysis and showed a durable response to ICI. The second patient remained dialysis-dependent post-ICI treatment and was readmitted with allograft complications leading to his eventual death.

**Teaching Points:**

This study underscores the complexity of managing KT recipients diagnosed with malignancy and receiving ICIs. The balance between immunosuppression reduction to treat malignancy and preventing allograft rejection presents a significant challenge. Key considerations include the risk of acute allograft rejection and patient-centered decision-making. These cases highlight the need for further research to develop evidence-based guidelines for managing this patient population. In addition, the patient perspective in this study highlights the importance of careful risk-benefit analysis and the impact of treatment decisions on patient-focused outcomes.

## Résumé

**Contexte:**

Les receveurs d’une greffe rénale (RGR) présentent un risque accru de cancer en raison de l’immunosuppression chronique. L’utilisation émergente des inhibiteurs du point de contrôle immunitaire (IPCI) s’est avérée un développement prometteur pour traiter les cancers, mais elle ajoute à la complexité de la gestion de l’immunosuppression chez les RGR. Cette étude décrit deux cas de rejet aigu chez des RGR après l’initiation des IPCI et discute du difficile équilibre entre le traitement du cancer et une immunosuppression adéquate.

**Présentation des cas:**

Le premier patient est un homme de 44 ans ayant reçu un diagnostic de carcinome à cellules rénales métastatique qui a présenté une insuffisance rénale aiguë six jours après l’initiation des IPCI. Le second patient est un homme de 73 ans ayant reçu un diagnostic de carcinome épidermoïde qui a présenté une insuffisance rénale aiguë deux semaines après l’initiation des IPCI.

**Diagnostic:**

Dans les deux cas, le rejet aigu a été diagnostiqué à la suite de la réduction de l’immunosuppression et de l’initiation des IPCI.

**Intervention:**

Les deux patients ont reçu une dose plus élevée de stéroïdes sans amélioration de la fonction du greffon. Le premier patient a subi une néphrectomie retardée du greffon en raison de complications de rejet aigu, ce qui n’a pas été le second patient.

**Résultats:**

Le premier patient a subi des complications, notamment des saignements périopératoires qui ont nécessité de multiples opérations, mais s’est finalement stabilisé avec l’hémodialyse et a montré une réponse durable aux IPCI. Le second patient est demeuré sous dialyse après le traitement aux IPCI et a été réadmis avec des complications de l’allogreffe qui ont entraîné son décès.

**Enseignements tirés:**

Cette étude souligne la complexité de la prise en charge des RGR qui reçoivent un diagnostic de tumeur maligne et des traitements par IPCI. L’équilibre entre la réduction de l’immunosuppression pour traiter le cancer et la prévention du rejet de l’allogreffe constitue un important défi. Le risque de rejet aigu de l’allogreffe et la prise de décision centrée sur le patient sont des éléments clés à prendre en compte. Ces deux cas soulignent la nécessité de poursuivre les recherches afin d’élaborer des lignes directrices fondées sur des données probantes pour la gestion de cette population de patients. De plus, le point de vue du patient dans cette étude met en évidence l’importance d’analyser rigoureusement le rapport risques-bénéfices et l’impact des décisions de traitement sur les résultats axés sur le patient.

## Introduction

Kidney transplant (KT) patients are at an increased risk of developing malignancies, such as skin cancer, lymphoma, and kidney cancer, with an estimated 20% absolute risk of malignancy following 10 years of chronic immunosuppression.^1^ This is in part due to the immunosuppressive agents used to prevent allograft rejection that concurrently reduce the immune system’s ability to suppress the growth of cancerous cells. For this reason, treatment of malignancy in KT patients often involves modification, reduction, or cessation of immunosuppression, which in turn increases the risk of allograft rejection.^2,3^

Treating malignancy in KT patients has been further complicated by the development of newer immunotherapy agents used to treat cancer, such as the immune checkpoint inhibitors (ICIs). These agents work by blocking immune regulatory pathways, leading to stimulation of the immune system and promoting destruction of cancer cells.^4^ This immune stimulation, in conjunction with cessation of immunosuppression in KT, may lead to a greater risk of acute allograft rejection and potentially graft-related complications.^5^

The impact of ICI therapy on graft function and subsequent graft complications in KT recipients is not well understood, and here we describe 2 cases of acute renal allograft rejection following cessation of immunosuppression and initiation of immunotherapy.

## The first patient

### Presenting Concerns

A 44-year-old male KT recipient was diagnosed with metastatic clear cell renal cell carcinoma following an incidental finding of an 8-cm mass on ultrasound of his renal allograft. The case was discussed at genitourinary cancer site team rounds, where it was felt that surgical resection with curative intent was not achievable and that upfront cytoreductive nephrectomy would not be a favorable approach in light of rapidly progressive disease and the need for upfront systemic therapy. Transplant-related immunosuppression was stopped and he was started on ICI immunotherapy (nivolumab and ipilimumab). One month following cessation of immunosuppression, and 6 days after starting ICI, he presented to the emergency department (ED) with a 1-day history of fever. A chronological timeline is described in Table 1.

**Table 1.** Timeline—The first patient.

Time to admission	Event
−20 years	Living-related kidney transplant for end-stage kidney disease secondary to congenital oligomeganephronia
−106 days	Ultrasound shows 8 cm renal mass
−97 days	MRI suspicious for malignancy
−31 days	CTA supports diagnosis of malignancy
−29 days	Admitted to nephrology for workup of mass. Kidney mass biopsy shows renal cell carcinoma, clear cell type. Medical oncology consulted. All immunosuppression stopped
−6 days	Started nivolumab and ipilimumab
Admission	Presented to ED with fever, found to have acute on chronic kidney injury. Admitted to nephrology with suspicion of acute allograft rejection. Started on prednisone 20 mg daily
+1 day	CT of the abdomen and pelvis showed increased size of the renal mass and transplant renal vein tumor thrombus
+6 days	Seen by transplant surgery. Planned for graft nephrectomy to prevent graft complications
+8 days	Kidney function continued to deteriorate, started on hemodialysis
+13 days	Underwent graft nephrectomy
+15 days	Returned to operating room for post-operative bleeding, abdomen left open
+17 days	Returned to operating room to close abdomen
+32 days	Discharged home
+2 years	Seen in medical oncology clinic. CT shows no evidence of recurrence after completing 18 cycles of maintenance nivolumab

MRI = magnetic resonance imaging; CTA = computed tomography angiography; ED = emergency department; CT = computed tomography.

### Clinical Findings

Past medical history was otherwise significant for end-stage kidney disease (ESKD) secondary to congenital oligomeganephronia for which he received a living-related KT 20 years prior to admission. In the year prior to his cancer diagnosis, he had a baseline serum creatinine concentration of 150 to 180 μmol/L on stable maintenance immunosuppression with cyclosporine 5 mg twice a day and prednisone 5 mg daily. He also had hypertension, dyslipidemia, gout, hypothyroidism, and experienced a pulmonary embolism for which he was anticoagulated. In response to his new cancer diagnosis, he stopped prednisone and cyclosporine 29 days prior to admission and received his first doses of nivolumab and ipilimumab 6 days prior to admission. When he presented to the ED, medications included allopurinol, diltiazem, rosuvastatin, rivaroxaban, and sennosides.

Examination revealed tenderness over the site of his kidney graft and sinus tachycardia with a heart rate of 146 beats per minute. Otherwise, vitals were stable and physical examination was unremarkable. Initial laboratory investigations revealed a serum creatinine concentration of 275 μmol/L (171 μmol/L 2 weeks prior). Urinalysis showed 3.0 g/L protein and a small amount of blood. Urine cultures and blood cultures were negative. There was no laboratory evidence of tumor lysis syndrome. Chest x-ray was unremarkable.

### Diagnostic Focus and Assessment

Additional investigation in hospital included a computed tomography (CT) of the abdomen and pelvis, which showed minimal interval increase in the size of the left KT mass, transplant renal vein findings consistent with tumor thrombus, and an interval increase in known retroperitoneal lymphadenopathy. A subsequent ultrasound confirmed a near occlusive thrombus in the transplant renal vein. Given the sudden decrease in kidney function in the context of recently discontinuing immunosuppression, starting immunotherapy, and the presence of graft-site tenderness, his presentation was felt to be most suspicious for acute allograft rejection, with a possible component of renal vein thrombosis contributing, and he was started on prednisone 20 mg daily. He was also given intravenous (IV) fluids, broad spectrum antibiotics, and was started on a heparin infusion in place of rivaroxaban. During his admission, he developed a fever, hematuria, and dysuria and the question of a urinary tract infection was raised, but ultimately repeated urine cultures were negative. His kidney function continued to deteriorate, with a serum creatinine concentration progressively rising from 275 μmol/L to 677 μmol/L over a week and he was started on hemodialysis.

### Therapeutic Focus and Assessment

Given the suspicion for acute allograft rejection, the focus of discussion surrounded graft nephrectomy to avoid further complications. The case was discussed between the admitting nephrology team and the transplant surgery team. Ultimately it was felt that, while nephrectomy was indicated, the graft was likely significantly inflamed, making nephrectomy high risk at that time. Surgery was delayed for this reason, and graft nephrectomy subsequently took place on post-admission day 13. The explanted graft was examined for tumor characteristics and surgical margins, but tissue was not examined for evidence of rejection.

On post-operative day 2, the patient was brought back to the operating room (OR) for a progressive drop in hemoglobin suspicious for bleeding. In the OR, approximately 900 cc of blood was evacuated from his abdomen, although no clear source of bleeding was identified, and his abdomen was left open. Two days later, he returned to the OR again for removal of packing and abdominal closure. He subsequently stabilized clinically and was discharged home from hospital on post-admission day 31.

### Follow-up and Outcomes

Following discharge, the patient continued maintenance nivolumab for 18 cycles with a durable response to treatment. He continued hemodialysis and remained stable from a malignancy perspective with no radiographic evidence of recurrence 2 years post-nephrectomy.

## The second patient

### Presenting Concerns

A 73-year-old male KT recipient was found to have a highly infiltrative squamous cell carcinoma of the right temple for which he underwent multiple surgical excisions and radiation treatments, in addition to a reduction of tacrolimus. Despite this, there was a recurrence with evidence of bony invasion, therefore transplant-related immunosuppressants, specifically tacrolimus and mycophenolate mofetil (MMF), were stopped and he was started on ICI immunotherapy (cemiplimab). Three weeks following cessation of immunosuppression, and 2 weeks after starting ICI, he presented to the ED with a 4-day history of nausea, vomiting, and diarrhea, followed by 2 days of generalized weakness and decreased urine output. A chronological timeline is described in Table 2.

**Table 2.** Timeline—The second patient.

Time to admission	Event
−4 years	Underwent deceased donor kidney transplant for end-stage kidney disease secondary to hypertension and IgM glomerulonephritis
−629 days	Facial SCC diagnosed. Underwent excision of right temple lesion
−485 days	Tacrolimus dose reduced
−542 to 390 days	Underwent multiple resections of right SCC
−360 days	Started radiation treatment
−149 days	CT scan showed local recurrence
−80 days	MRI showed bony invasion
−57 days	Seen in renal transplant clinic. Stable graft function on standard immunosuppression. Decision to proceed with immunotherapy in context of recurrence of SCC. Mycophenolate and tacrolimus doses reduced with plans to stop both prior to immunotherapy. Counseled this would likely result in rejection
−23 days	Mycophenolate and tacrolimus stopped
−17 days	Cemiplimab started
Admission	Presented to ED with acute kidney injury. Admitted to nephrology with suspected acute allograft rejection. Started on intravenous steroids
+2 days	Kidney function continued to deteriorate and started on hemodialysis
+4 days	Seen by transplant surgery team. No graft nephrectomy felt to be indicated at that time
+8 days	Discharged home
+55 days	Received last dose of cemiplimab
+58 days	Admission to nephrology with renal graft infection
+70 days	Carcinoma of bladder diagnosed on cystoscopy
+78 days	Discharged home for palliation

SCC = squamous cell carcinoma; CT = computed tomography; MRI = magnetic resonance imaging; ED = emergency department.

### Clinical Findings

Past medical history was otherwise significant for ESKD secondary to hypertension and IgM glomerulonephritis for which he received a deceased donor KT 4 years prior to admission. He was stable on maintenance immunosuppression with tacrolimus 1.5 mg daily, MMF 750 mg twice daily, and prednisone 5 mg daily and had a baseline serum creatinine concentration of approximately 90 to 110 μmol/L. He also had a history of hypertension, dyslipidemia, gout, gastroesophageal reflux disease, colonic polyps, diverticulosis, hemorrhoids, and tonsillectomy. He had stopped MMF and tacrolimus 23 days prior to admission and had his first dose of cemiplimab 17 days prior to admission. At the time of presentation to the ED, medications included prednisone, amlodipine, propranolol, atorvastatin, allopurinol, and pantoprazole.

Examination revealed mild lower limb edema and mild tenderness over the site of his renal graft. Otherwise, his examination was within normal limits and he was vitally stable. Initial laboratory investigations revealed a serum creatinine concentration of 651 μmol/L (110 μmol/L 2 weeks prior). Urinalysis showed 3.0 g/L protein and 50 to 100 red blood cells/high powered field. There was no laboratory evidence of tumor lysis syndrome.

### Diagnostic Focus and Assessment

Further investigation included an abdominal CT that showed marked enlargement and perinephric stranding of the right transplanted kidney, associated with pelvic free fluid and inflammatory alteration of the bladder, suggestive of an inflammatory/infectious etiology, possible autoimmune reaction, or rejection. Given the rapid decline in kidney function, graft-site tenderness, and CT findings occurring in the context of recent cessation of immunosuppression and initiation of immunotherapy, acute allograft rejection was suspected, with a possible component of immune-mediated toxicity or prerenal injury secondary to gastrointestinal losses. He was given 80 mg of IV solumedrol in the ED and admitted to the nephrology service. During his admission, he was reviewed by the medical oncology team and it was felt that his diarrhea may be related to immune-mediated colitis. His renal function continued to decline despite volume repletion, with his serum creatinine concentration increasing to 899 μmol/L and urine output dropping to 225 mL/day. He was started on hemodialysis on post-admission day 2.

### Therapeutic Focus and Assessment

Given the suspicion for acute allograft rejection and possible immune-mediated renal toxicity contributing to deterioration of his renal function in addition to possible immune-mediated colitis, high dose solumedrol was continued on admission at a dose of 2 mg/kg. Surgery was consulted to consider the role of graft nephrectomy, but it was felt to be unnecessary at that time in the absence of graft-site tenderness or hematuria. During his admission, he remained stable on hemodialysis and IV steroids were reduced. There was no evidence of renal recovery and he was discharged home on post-admission day 7 with a prednisone taper (60 mg daily, tapering by 10 mg every 5 days) and ongoing hemodialysis.

### Follow-up and Outcomes

Following discharge, the patient remained dialysis-dependent and completed his steroid taper. He continued immunotherapy, receiving his last dose 2 weeks after discharge. Three weeks after discharge, he was readmitted to nephrology with a renal allograft infection complicated by bacteremia. Intravenous antibiotics were initiated and a percutaneous drain was placed. During this admission, he was found to have gross hematuria and CT findings of a bladder mass and therefore underwent cystoscopy that showed a new diagnosis of bladder carcinoma. In the context of 2 active malignancies, graft infection, and overall frailty, he was discharged home for palliation and subsequently passed away.

### Discussion

Malignancy represents a significant complication for KT recipients and is a major limiting factor for life expectancy in this population.^6,7^ Given that the immunosuppression necessary for graft survival contributes to an increased risk of cancer in these patients, management of immunosuppression and cancer therapeutics represents a substantial challenge, lacking evidence-based guidelines.^2^

Immune checkpoint inhibitors are a growing class of anti-cancer agents being used with increasing frequency to treat a variety of cancers.^7^ These agents work by promoting immune system activation with resulting destruction of malignant cells. This is based on the premise that the immune system contains a number of “checkpoints” that are critical for self-tolerance and regulating the duration and amplitude of immune responses.^8^ It is now apparent that certain malignancies are able to utilize these pathways as a mechanism of immune resistance.^8^ The fundamental principle underpinning ICI therapy is that by targeting these pathways, and thus blocking these immune checkpoints, we are able to enhance antitumor immunity. In contrast, medications routinely used to prevent allograft rejection in KT, such as MMF and calcineurin inhibitors, are used to suppress the immune system. These immunosuppressants have been associated with an increased incidence of malignancy due to suppression of the antitumor effects of the immune system.^9,10^

Solid organ transplant recipients have traditionally been excluded from ICI randomized control trials, further weakening the evidence available to guide decision-making.^11^ Given the conflicting indications for immunosuppression and immune activation in these patients, as well as the paucity of data available, the decision-making process for patients and physicians is complex. There have been several case reports, case series, and more recently systematic reviews published describing the outcomes for KT recipients receiving ICIs, reporting kidney allograft rejection rates of 35% to 45% on ICI, however, with limited Canadian context, the current study adds to this growing body of literature.^5,11-15^

The implications of acute renal allograft rejection and its associated complications on patient morbidity and mortality are important factors to consider in the initiation of ICI in KT recipients. Here, we describe 2 cases of suspected acute rejection following initiation of ICI, acknowledging these were clinical diagnoses and not able to be confirmed by histologic examination, and the differential diagnosis included contribution from renal vein thrombosis (the first patient) and/or prerenal injury (the second patient). These cases highlight the need to carefully consider the approach to managing malignancy in KT recipients and further prompt clinicians to seek standardized, evidence-based practices to guide decision-making. There are a variety of factors that may affect a patient’s decision-making process in these circumstances, including concerns of allograft rejection, the need for kidney replacement therapy, the impact on quality of life, life expectancy, and prognosis of malignancy. A robust and individualized discussion around these factors is a reasonable first step for these challenging cases.

The role, if any, of pre-emptive graft nephrectomy in patients initiating ICI, and how this may relate to a more nuanced risk discussion with patients, has not been well discussed in the literature. In the cases described here, both patients experienced suspected renal allograft rejection and resulting life-threatening complications. A delayed graft nephrectomy was performed in the first patient, which was complicated by a post-operative bleed and multiple repeat surgeries. The patient in the second patient did not undergo a graft nephrectomy, but subsequently experienced a serious allograft infection and bacteremia, contributing to his eventual death. While there is a paucity of data to recommend pre-emptive graft nephrectomy, it represents an area for further research, as identifying patients at high risk for rejection and subsequent complications following ICI initiation, may help guide a more informed risk/benefit discussion of this intervention.

Alternative management strategies for these patients are being explored, including continuation of baseline immunosuppression, increased doses of prednisone, and addition of mTORi (mammalian target of rapamycin inhibitor). Currently, management of baseline immunosuppressive medications at the time of diagnosis of malignancy is variable, with most centers electing to reduce immunosuppression.^15^ Recently, a single-arm, phase 1 study of 17 KT recipients being treated with ICI with no modification of their baseline immunosuppression regimen demonstrated a graft rejection rate of only 12% and a oncologic response rate (complete and partial) of 53%.^16^ This provides support that continuing baseline immunosuppression may not adversely impact oncologic outcomes and may improve rejection rates compared to other reports, but the conclusions that can be drawn from this small study are limited. Use of increased doses of prednisone has been examined in this population as well, with a small case series demonstrating that a short course of prednisone (20-40 mg/day over 1-2 weeks) yielded fewer episodes of rejection,^17^ but a second study showed no difference in rejection rates with higher prednisone.^15^ The role of converting to mTORi has been examined as well, with a reports of a reduced risk of rejection with mTORi use.^18^ Ultimately, while there are a number of novel treatment regimens being investigated, currently, data to guide the management of these patients are lacking, and further study is needed.

Management of KT recipients undergoing ICI poses a significant challenge due to the competing needs for immunosuppression to prevent graft rejection and immune activation for tumor suppression. Evidence-based guidelines are lacking regarding the management of these patients and clinical decision-making remains challenging. As such, there is a need for individualized treatment plans for patients starting ICIs, considering factors, such as risk of allograft rejection, malignancy prognosis, and patient preferences, underscoring the need for further research to establish standardized practices.

## Patient Perspective

The first patient: “[. . .] what happened was exactly what [my urologist] had thought and I had a hot rejection and that made the nephrectomy a lot more dangerous. [. . .] the dates and times constantly changed as to when the nephrectomy would take place as they were still on the fence and undecided as to the risk/reward and the benefits of having it removed. [. . .] I thought the level of care was wonderful. I’ve been cancer free now since late 2019. I play golf 2-3 times per week, I coach my son’s ball team, and I umpire ball, so I lead a pretty active life.”
